# Analysis of Ecological Quality of the Environment and Influencing Factors in China during 2005–2010

**DOI:** 10.3390/ijerph110201673

**Published:** 2014-01-30

**Authors:** Shi-Xin Wang, Yao Yao, Yi Zhou

**Affiliations:** 1 State Key Laboratory of Remote Sensing Science, Institute of Remote Sensing and Digital Earth, Chinese Academy of Sciences, Beijing 100101, China; E-Mails: wsx@irsa.ac.cn (S.-X.W.); zhouyi@irsa.ac.cn (Y.Z.); 2 Graduate School of Chinese Academy of Sciences, Beijing 10049, China

**Keywords:** ecological quality of environment, remote sensing and GIS, Eco-environmental Quality Index (EQI), ecological environmental change

## Abstract

Since the twentieth century, China has been facing various kinds of environmental problems. It is necessary to evaluate and analyze the ecological status of the environment over China, which is of great importance for environmental protection measures. In this article, an Eco-environmental Quality Index (EQI) model is established using national remote sensing land-use data, NDVI data from MODIS and other statistical data. The model is used to evaluate the ecological status over China during 2005, 2008 and 2010, and spatial and temporal variations in EQI are analyzed during the period 2005–2010. We also discuss important factors affecting ecological quality, with special emphasis on meteorological conditions (including rainfall and sunshine duration) and anthropogenic factors (including normalized population and gross domestic product densities). The results show that, EQIs in northwestern China are generally lower than those in the southeast of the country, presenting a ladder-like distribution. There is significant correlation between EQI, rainfall and sunshine duration. Population density and GDP also have some relation to EQI. On the whole, the environmental quality results showed little variation during 2005–2010, with national average EQIs of 54.86, 55.07 and 54.43 in 2005, 2008 and 2010, respectively. During 2005–2010, differences in EQI were observed at the local level, but those at the provincial level were small.

## 1. Introduction

Since the twentieth century, with rapid population growth and the acceleration of industrialization and urbanization, ecological and environmental problems have been paid close attention and have become issues of global concern. As a developing country with the largest population and the third-largest land area in the World, China has been facing especially serious ecological and environmental problems. Global warming [1,2,3], soil erosion [4], biodiversity loss [5], air pollution, water pollution [6] and water shortages are presently the major environmental problems in China. Based on comprehensive consideration of natural environment, socioeconomic status and various kinds of environmental problems, it is necessary to evaluate and analyze the environment status over China.

Much progress has been made in the area of research into ecological environmental problems. At the end of the 1980s, the Organisation for Economic Co-operation and Development (OECD) developed a Pressure–State–Response Framework model (PSR) that provided a logical basis for assessment of ecosystem health [7]. The model reflects the relationships between Nature, society and economic factors within an ecosystem from the perspective of the combination of economic development and environmental protection, and has been widely applied in ecological and environment assessment. Subsequently, the U.S. Environmental Protection Agency, the Commission on Sustainable Development and the European Environment Agency developed improved forms of the PSR model, which have been used to study the ecological and environmental qualities of many regions [8,9,10]. Since the concept of sustainable development was proposed, researchers commonly view ecological and environmental assessment within the framework of sustainable development, and two evaluation indexes of ecological environment have been proposed: Ecological Footprint [11] and Environmental Sustainability Index [12]. Fricker and Vurren [13,14] calculated the ecological footprints of New Zealand, the Kingdom of Bhutan and The Netherlands. Matthew *et al*. assessed and compared the urban ecosystem status of 20 American cities via an improved ecological footprint method [15]. Xu *et al*. calculated the ecological footprints of some provinces of China based on statistical data for 1999, noting that in most provinces, the ecological footprints exceeded the ecosystem carrying capacity [16,17].

A review of the relevant literature on ecological environmental assessment identified two shortcomings in prior studies. The first is that previous environmental assessment methodologies mainly focused on ecological impact assessment, ecological risk assessment and ecological fragility assessment, whereas few studies conducted a comprehensive assessment of the present ecological environmental situation. For example, Trevisan used the Nonpoint-Source Agricultural Hazard Index (NPSAHI) with GIS to analyze the influence of agricultural practices on ecological environment in the province of Cremona in Italy [18]. Sun *et al*. evaluated the ecological fragility of the Wuzhishan Nature Reserve by analyzing the soil, plants and ecosystem [19]. Another drawback is that most previous studies examined specific regions, and so the study areas are usually at the province or county level. Musacchio *et al*. assessed and made a planning on wetland and farmland ecosystems in Texas using related indexes of landscape ecology [20]. Manson explored land-use/cover changes and assessed the effects of ecological structure in the southern Yucatán peninsular region (SYPR) of Mexico using dynamic spatial simulation [21]. Li *et al*. evaluated eco-environmental vulnerability in mountainous regions using remote sensing and GIS via analytic hierarchy process and comprehensive evaluation method [22]. Few studies in the literature have reported on large-scale regions.

The State Environmental Protection Administration of China (SEPA) published its “Technical Criterion for Eco-environmental Status Evaluation” [23] in 2006. This established an Eco-environmental Quality Index (EQI) model to evaluate environment status in China via Biological Abundance Index (BAI), Vegetation Index (VI), Water Network Density Index (WNDI), Land Degradation Index (LDI) and Pollution Index (PI), considering ecological and environmental characteristics relevant to China. This method has been widely used to study the environment status of specific regions in China. Wei *et al*. evaluated the EQI of Fujian using land-use data for 2009 [24] and Meng *et al*. evaluated the EQI of Kenli County using China–Brazil Earth Resources Satellite (CBERS) multi-spectral imagery [25].

Therefore, in the present study we selected five indicators by which to evaluate ecological and environmental problems and status over China (excluding Taiwan, Hong Kong and Macao) in 2005, 2008 and 2010. This article also discusses the spatial and temporal distributions and factors influencing the Chinese environment. 

## 2. Materials and Methods

### 2.1. Materials

The present study uses several data sets to evaluate EQI. These include land-use monitoring data, MODIS NDVI products, quantitative water resources, soil erosion, pollutant emission, annual rainfall, annual number of sunshine hours, population density, and GDP density.

The 1:100,000 land-use data (1,000 m resolution) over China in 2005, 2008 and 2010, which are interpreted by Landsat TM data, are supported by the Institute of Remote Sensing and Digital Earth at the Chinese Academy of Sciences. The data are projected to an Albers Conical Equal Area projection. The data use six classifications and 25 sub-classifications, including forest, grassland, farmland, water body, constructive land and unused land. The average classification accuracy is 95.41% [26].

The MODIS NDVI product (MOD13A2, 16-day, 1,000 m resolution) during January to December of 2005, 2008 and 2010 were downloaded from NASA [27]. The maximum NDVI values in 2005, 2008 and 2010 were extracted from the 16-day data using the maximum-value composite procedure (MVC) to evaluate EQI [28,29]. The MVC method can reduce the noise caused by cloud cover. To avoid inflating the Vegetation Index, we set the value of snow/water/clouds (NDVI < 0) to 0, treating these areas as bare soil.

Water resource data comprise the total quantity of groundwater and surface water. Water resource quantity data (1 km^2^ grid) are supported by the Institute of Remote Sensing and Digital Earth, Chinese Academy of Sciences.

The soil erosion data were produced by the Ministry of Water Resources in 1995 from a combination of ground monitoring and remote sensing, and were obtained from the Data Sharing Infrastructure of Earth System Science, China [30]. 

The pollutant emission data are evaluated with reference to pollutant statistics obtained from the “China Statistical Yearbook on Environment” (CSYE) [31,32,33]. The pollutant data for Taiwan, Hong Kong and Macao were not considered, and these areas are therefore excluded from the study.

Annual rainfall and sunshine hours for 2005 were obtained from the China Meteorological Data Sharing Service System [34]. The original station observation data are supported by 756 meteorological observatories across China. The rainfall and sunshine hours (1,000 m grid) are interpolated by a Kriging Sphere Model [35].

The population density data (1,000 m resolution) are evaluated by population statistics and DMSP/OLS night-time light images [36]. The GDP density grid data (1,000 m resolution) over China in 2005 are corrected by land-use monitoring data and DMSP/OLS night-time light images, based on GDP statistics [37].

### 2.2. Methods

This study evaluates ecological qualities of environment over China in 2005, 2008 and 2010. The study area comprises the entire Chinese mainland, which presents features such as extensive areas, complex land types, poor accuracy of statistical data, and so on. As a result, conventional statistical methods such as variance and standard deviation are unsatisfactory. Therefore, the ecological environment quality indices were evaluated via a synthetic index based on “Technical Criterion for Eco-environment Status Evaluation” with an analytic hierarchy process and comprehensive evaluation method. The evaluation model consists of five indices: Biological Abundance Index (BAI), Vegetation Index (VI), Water Network Density Index (WNDI), Land Degradation Index (LDI) and Pollution Index (PI) [38]. The BAI is composed of forest, grassland, farmland and unused land derived from land-use data. The VI, which shows the vegetation cover over China, is calculated from NDVI data. The LDI describes water erosion, wind erosion and freeze-thaw erosion. The WNDI shows the distribution of the water network over China, based on a comprehensive consideration of water resources, including rivers and lakes. The PI measures environmental pollution over China through the emissions of SO_2_, chemical oxygen demand (COD) and solid waste.

Due to differences in parameter dimensions and magnitudes, the quantitative indexes are first normalized prior to calculation, as shown in Equation (1):

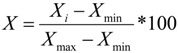
(1)


Here, *X* is the result after normalization, *X*_max_ is the maximum value and *X*_min_ is the minimum value.

After calculating the five indexes above, the final evaluation model is designed using the comprehensive index method [38]:

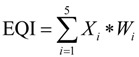
(2)


Here, EQI denotes the Eco-environmental Quality Index, *X*_i_ is the *i*th index and *W*_i_ is the weight of the *i*th index. The weight of each index can be seen in Table 1:

**Table 1:** The weight of each index in EQI.

Index	BAI	VI	WNDI	LDI	PI
Weight	0.25	0.2	0.2	0.2	0.15

## 3. EQI Results

### 3.1. Spatial Distribution Analysis

**Figure 1:**
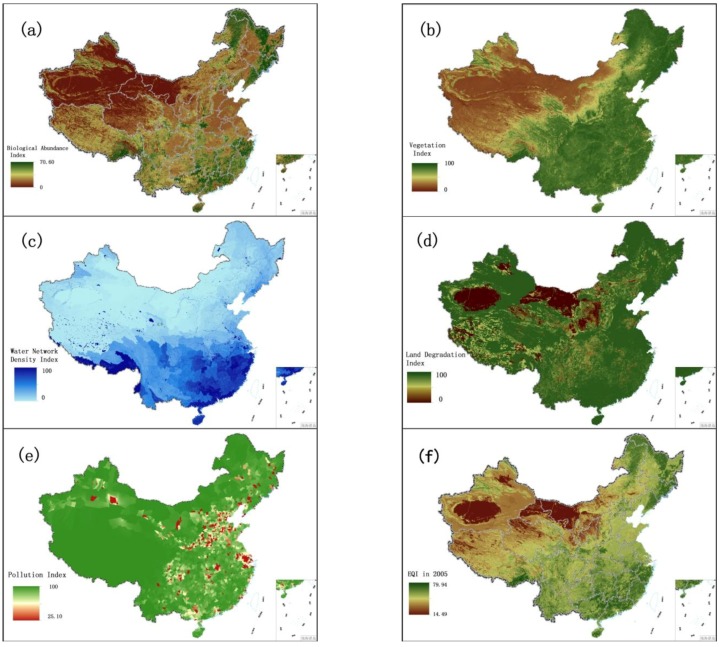
Ecological quality of environment over the Chinese mainland in 2005. (**a**) Biological Abundance Index; (**b**) Vegetation Index; (**c**) Water Network Density Index; (**d**) Land Degradation Index; (**e**) Pollution Index; (**f**) Eco-environmental Quality Index.

According to the ecological environment quality evaluation model, the national ecological environment quality maps for 2005 are as follows (Figure 1).

**Figure 2:**
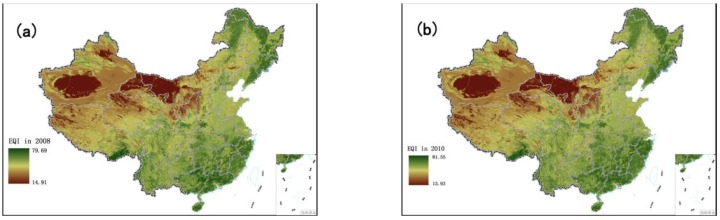
Ecological quality of environment over the Chinese mainland in 2008 and 2010. (**a**) EQI in 2008; (**b**) EQI in 2010.

The EQI results for 2008 and 2010 are evaluated via the same method (Figure 2):

**Figure 3:**
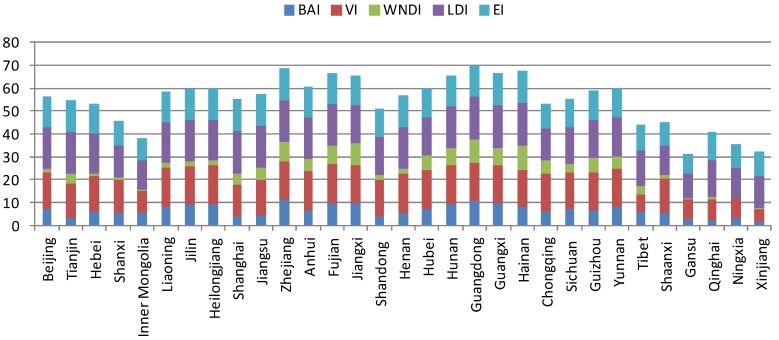
EQI distribution in each province.

According to Figure 1 and Figure 2, the spatial distributions of EQI in 2005, 2008 and 2010 are very similar. Figure 3 shows the distribution proportions of the average EQI value from 2005 to 2010 at the province level. As shown in the spatial distributions, the areas with the lowest EQI values are mainly located in the west and northwest regions of China (especially in Tarim Basin, Junggar Basin and the Inner Mongolia grassland desertification zone), which are the areas least suited to human settlement. As shown in Figure 3, water shortage, land degradation, low vegetation coverage and biological abundance are the dominant factors in these regions. Although degraded land is less marginal than desert and semi-desert, most parts of Xinjiang, Inner Mongolia, Gansu, Qinghai and Tibet are unsuitable for human development. Ecological environmental status in northern, central, southern and some parts of northeastern and northwestern China is intermediate, and is mainly distributed around the 400 mm rainfall isoline and the Aihui–Tengchong Line. The Aihui–Tengchong Line was proposed by Hu Huanyong, and divides China according to east and west populations and physical geography. West of the line, the main land use types are grassland, eremophytes and highlands, containing only about 5% of the human population. The low vegetation coverage and biological abundance of the provinces west of the line are greatly influenced by natural conditions. East of the Aihui–Tengchong Line, the geographic formations are composed of plain, water networks and hilly regions. The eastern part includes almost 95% of the population, so human factors play an important role in environment quality, causing low biological abundance index values in Henan, Hebei and Shandong, shown in Figure 3. Suitable regions are distributed in most regions of southeastern, southwestern and northeastern China. It is because that the forests are mainly in the southwest and northeast, and southeastern China is the region of highest water resources. Most of the regions with the highest EQI are in Guangdong, Zhejiang and the southern Himalaya. Figure 3 shows that the BAI, VI and WADI in these provinces are significantly higher than in other regions.

### 3.2. Factors Affecting Spatial Distributions

Ecological quality of environment is determined by a number of complex factors, primarily natural factors such as meteorological conditions, and anthropogenic factors like population density and economic development. In this article, we focus on influential meteorological and anthropogenic factors.

#### 3.2.1. Rainfall and Sunshine

Analysis of the correlations between EQI and several natural factors, including rainfall, sunshine, elevation and temperature, showed the strongest correlations were associated with rainfall and sunshine. This finding indicates that rainfall and sunshine are critical factors affecting the eco-environment. They play important roles in spatial distribution of biological diversity, and also affect land-use and economic development to some extent.

**Table 2:** Correlations of EQI with rainfall, sunshine.

	EQI	BAI	VI	WNDI	LDI	PI
**Rainfall**	0.831 ^**^	0.706 ^**^	0.665 ^**^	0.954 ^**^	0.591 ^**^	0.149
**Sunshine**	−0.731 ^**^	−0.57 ^**^	−0.703 ^**^	−0.811 ^**^	−0.434 ^*^	−0.128

** correlate significantly at 0.01 level; * correlate significantly at 0.05 level.

**Figure 4:**
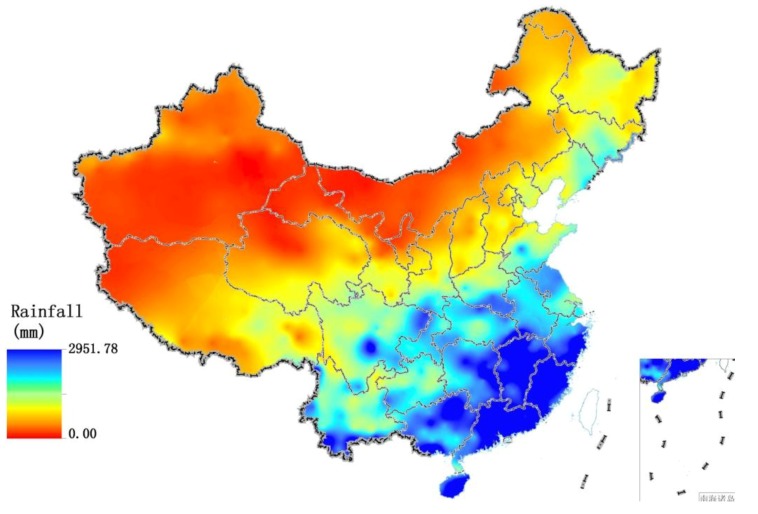
Spatial distribution of rainfall over China in 2005.

**Figure 5:**
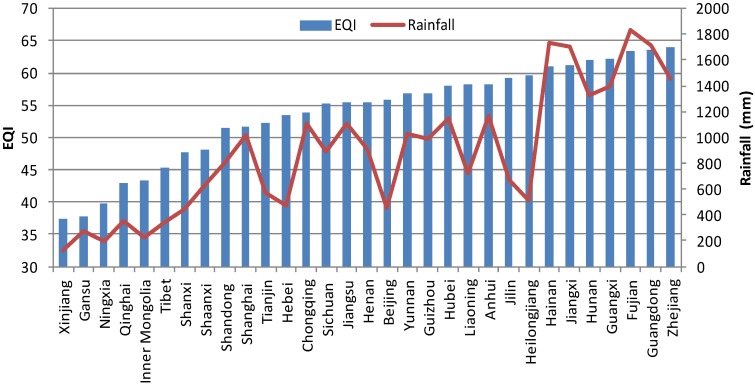
Comparison between rainfall and EQI over China at the provincial level.

**Figure 6:**
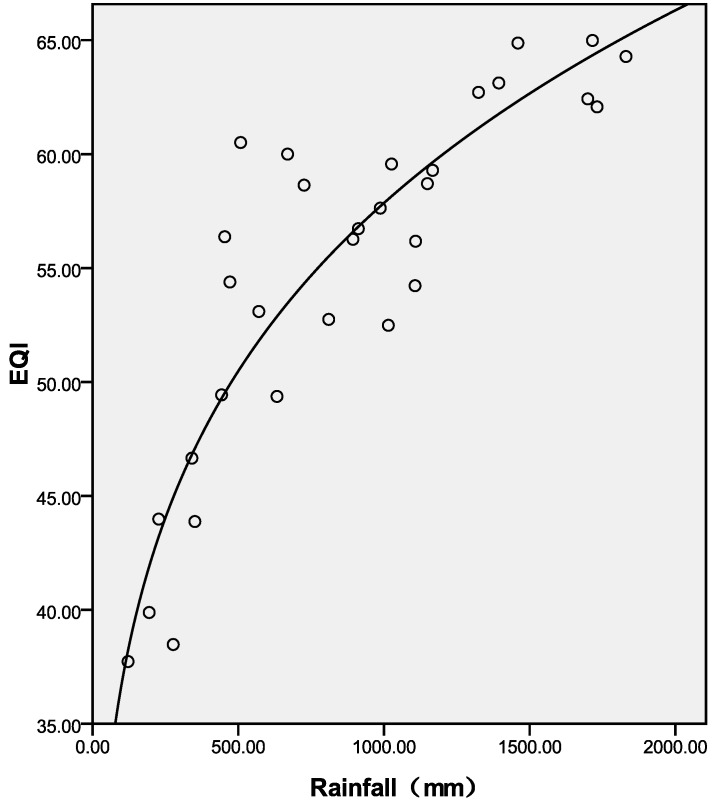
Best-fit curve for EQI and rainfall at the provincial level in 2005.

**Figure 7:**
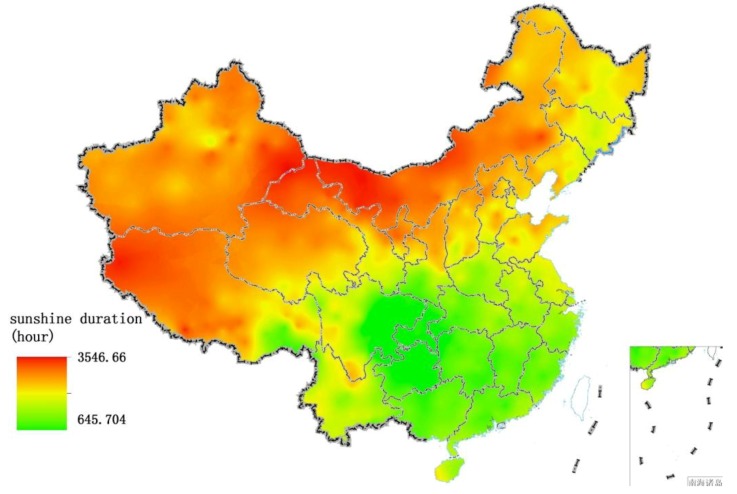
Spatial distribution of sunshine duration over China in 2005.

**Figure 8:**
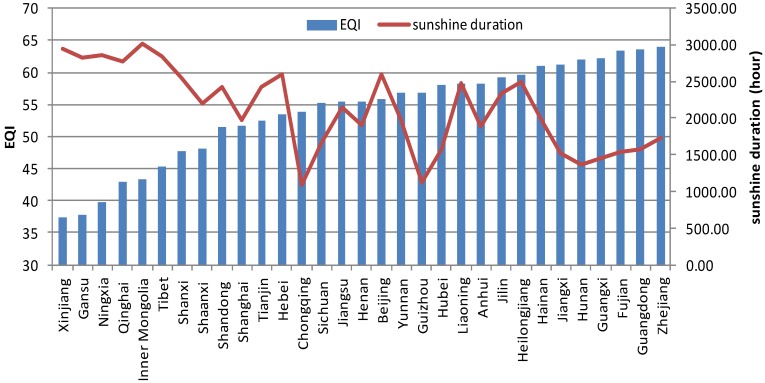
Comparison between sunshine duration and EQI over China at the provincial level.

**Figure 9:**
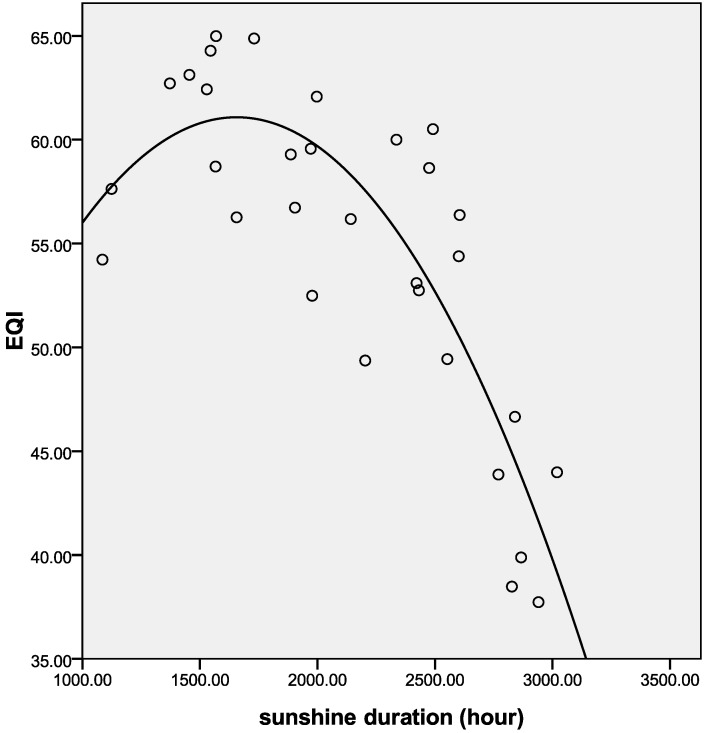
Best-fit curve for EQI and sunshine duration in 2005.

**Figure 10:**
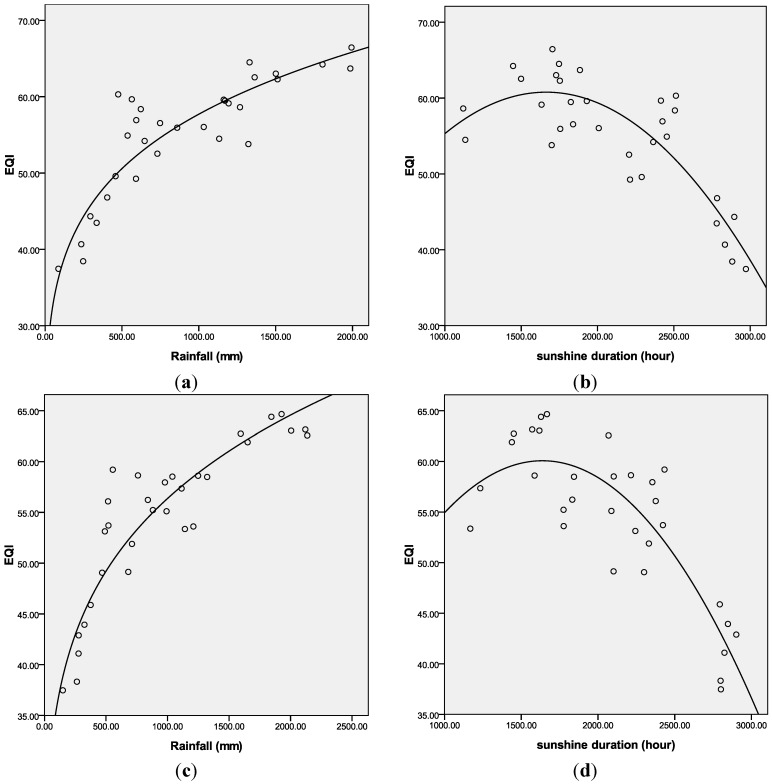
Best-fit curves for EQI, rainfall and sunshine duration in 2008 and 2010. (**a**) EQI and rainfall in 2008; (**b**) EQI and sunshine duration in 2008; (**c**) EQI and rainfall in 2010; (**d**) EQI and sunshine duration in 2010.

Table 2 shows the correlation coefficients between rainfall, sunshine and the indexes of EQI. BAI, VI, WNDI and LDI have significant positive correlations with rainfall (*p* < 0.01). The WNDI is directly affected by rainfall, so it has the strongest correlation. Vegetation conditions account for a large proportion of the correlation in BAI and VI, and therefore both of these indexes are also affected by rainfall. The LDI is calculated from soil erosion data, which mainly comprises erosion by freeze-thaw, water and wind mechanisms. Rainfall therefore has relatively small, but still significant correlation with LDI. PI is calculated from data on anthropogenic environmental pollution, and there is no significant correlation between rainfall and PI. BAI, VI, WNDI (*p* < 0.01) and LDI (*p* < 0.05) show significant positive correlations with sunshine. No significant correlation was found between sunshine and PI. Figure 4 shows the spatial distribution of rainfall over China in 2005, and Figure 5 and Figure 6 show the comparison between rainfall and EQI over China at the provincial level and the fitting curve. A strong positive correlation (0.831) was found between rainfall and EQI (*p* < 0.01). The best-fit result is expressed by a power function. With increasing rainfall, EQI is also increased, indicating higher quality of the environment. Figure 7 shows the spatial distribution of sunshine duration over China in 2005, and the comparison between sunshine duration; the associated provincial-level EQI and fitting curve are shown in Figure 8 and Figure 9. The correlation coefficient between sunshine duration and EQI is −0.734 (*p* < 0.01), meaning that longer sunshine duration corresponds with areas that display lower eco-environmental indices. The best-fit is expressed by a quadratic curve. Sunshine duration in Chongqing and Guizhou is very short because of the unique terrain and climate. However, the serious soil erosion caused by soil conditions results in low EQI in these regions. According to the relationships between rainfall, sunshine duration and EQI: in northern and western China, EQI is lower in areas with less rainfall and longer sunshine, while in southern and eastern China, EQI is higher with more rainfall and shorter sunshine. Figure 10 shows that the correlations between rainfall, sunshine and EQI are also significant in 2008 and 2010.

#### 3.2.2. Anthropogenic Factors

Besides meteorological conditions, ecological quality of environment depends on anthropogenic factors such as population and economic development [39]. In general, economic development closely relates to population density, and there is also interplay between ecological quality of environment, population and economy [40]. In the present study on the relationships between ecological quality of environment, population density and GDP density, the Chinese mainland is divided into eight economic regions in order to reduce the impact of natural factors.

**Figure 11:**
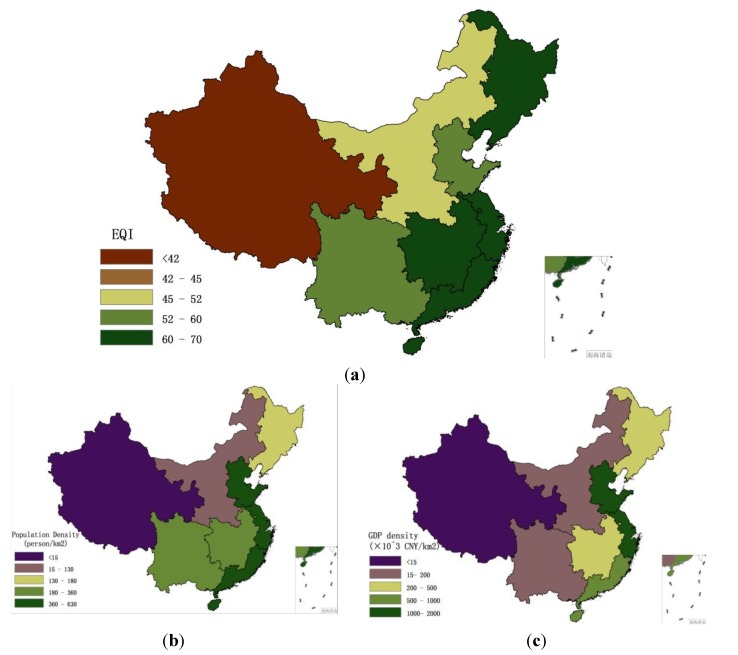
EQI, population density and GDP density in eight economic regions in 2005. (**a**) EQI; (**b**) population density; (**c**) GDP density.

**Figure 12:**
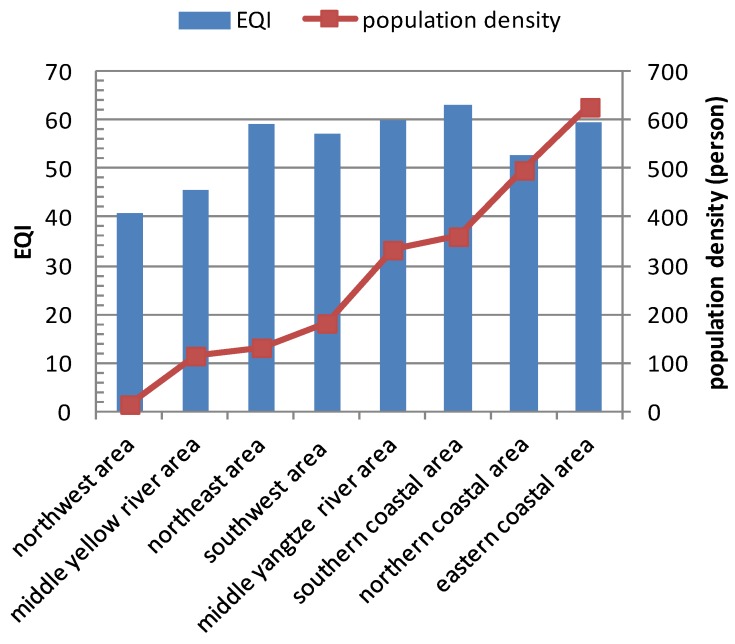
Relationship between EQI and population density in eight economic regions in 2005.

**Figure 13:**
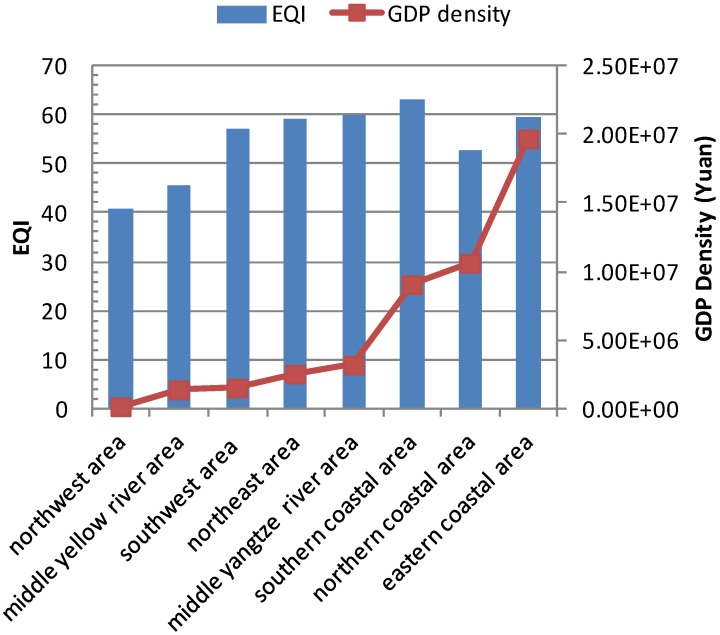
Relationship between EQI and GDP density in eight economic regions in 2005.

Figure 11 shows EQI, population density and GDP density within these eight economic regions in 2005. The relationship between EQI and population density is shown in Figure 12, and that between EQI and GDP density is shown in Figure 13. The results show that the spatial distribution of population density is generally consistent with that of GDP density in the eight economic regions. The population and GDP density are similar in the northeast area, southwest area and central Yellow River area, but the EQI result in the central Yellow River area is significantly lower than population and GDP. This is because the area has more serious water shortage and soil erosion problems than the other areas. When the 2005 EQIs of the eight economic regions are ranked according to population and GDP densities, some correlations are found. 

**Figure 14:**
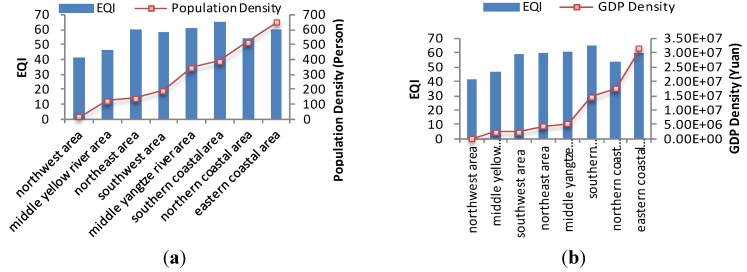
Relationship between EQI, population and GDP density in eight economic regions in 2008. (**a**) EQI and population; (**b**) EQI and GDP.

**Figure 15:**
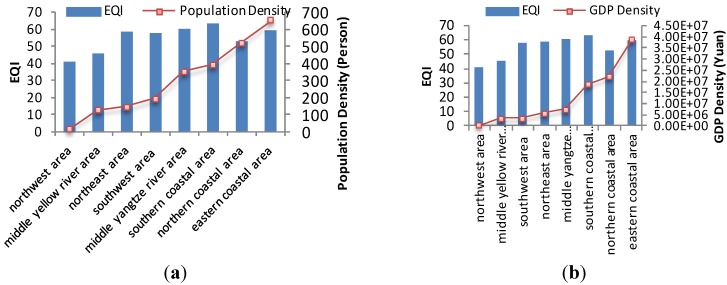
Relationship between EQI, population and GDP density in eight economic regions in 2010. (**a**) EQI and population; (**b**) EQI and GDP.

China is a large agricultural country undergoing rapid development, the majority of the population currently lives in rural areas, but the number of urban migrant workers has increased in recent years. According to the principle of maximization of benefits, there are shifts in the rural population from regions with poor environmental quality to locations with better environment because the latter places have better natural ecological conditions, developed industry and commerce, meaning higher income. As a result, better eco-environmental status is associated with higher population and aggregate GDP. However, a saturated population density exerts higher pressure on the environment, and can result in environmental deterioration. For example, the northern coastal area is the important economic center in northern China, and includes the Beijing–Tianjin–Hebei economic region. Many people are attracted to this region every year. Excessive exploitation of natural resources and lack of environmental protection lead to ecological and environmental deterioration. Although the population and GDP density in southern and eastern coastal areas are higher than other areas, the eco-environmental qualities are still better, because these areas have better meteorological conditions and are significantly richer in natural resources than other areas. Figure 14 and Figure 15 show the relationships between population density, GDP density and EQI within the eight economic regions in 2008 and 2010. The same trends are observed in 2008 and 2010.

## 4. Temporal Change of EQI during 2005–2010

**Figure 16:**
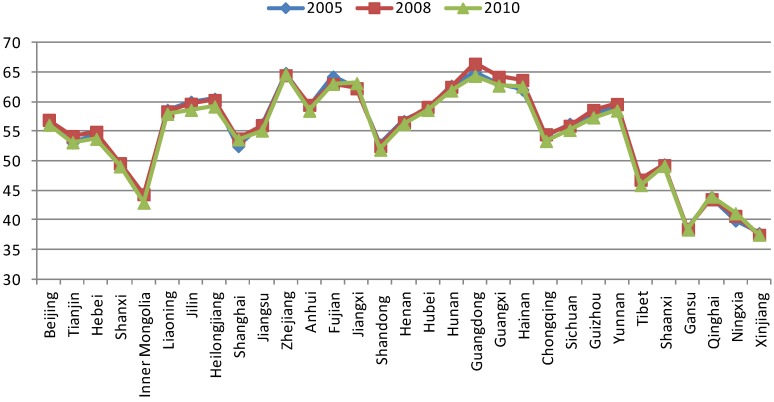
Average provincial EQI in China during 2005, 2008 and 2010.

**Figure 17:**
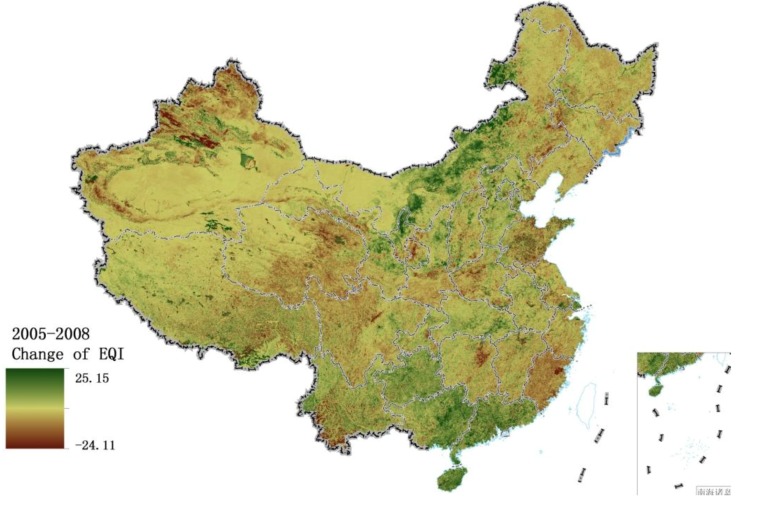
Change in EQI from 2005 to 2008.

**Figure 18:**
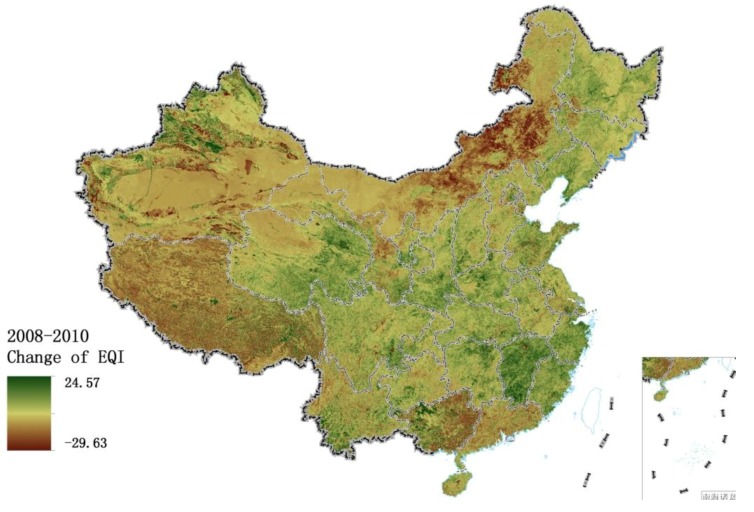
Change in EQI from 2008 to 2010.

Overall, ecological and environmental quality show little variation during 2005 to 2010: national average EQIs were 54.86, 55.07 and 54.43 in 2005, 2008 and 2010, respectively. Figure 16 shows little variation in the provincial average EQIs for 2005, 2008 and 2010, but local details can be seen from Figure 17 and Figure 18. 

**Figure 19:**
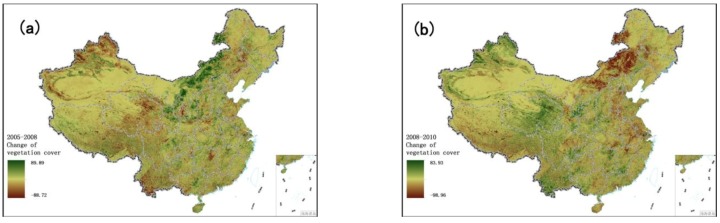
Change in vegetation cover over China during 2005–2010. (**a**) 2005 to 2008; (**b**) 2008 to 2010.

**Figure 20:**
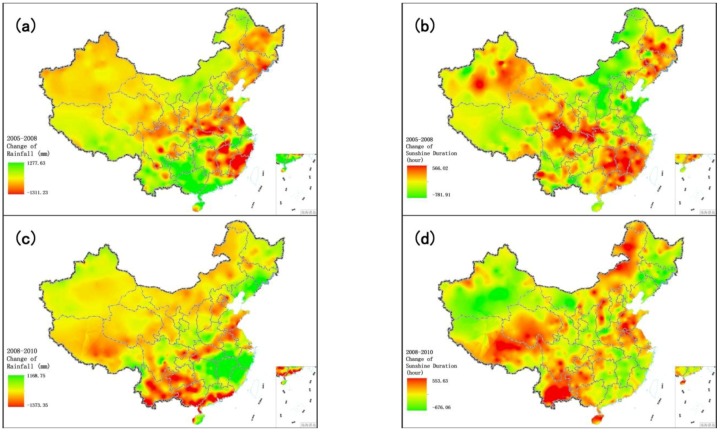
Change in meteorological conditions over China during 2005–2010. (**a**) Change in rainfall from 2005 to 2008; (**b**) Change in sunshine duration from 2005 to 2008; (**c**) Change in rainfall from 2008 to 2010; (**d**) Change of sunshine duration from 2008 to 2010.

As seen in Figure 17, the areas with significant improvement in EQI from 2005 to 2008 are mainly located in Yunnan, Guizhou, Guangdong, Guangxi, Hainan, northeastern Inner Mongolia and a few parts of western Xinjiang. Chapter 23 of “Guidelines of the Eleventh Five-Year Plan for National Economic and Social Development”, entitled ‘Protect and Remedy Natural Ecology’ recommended: afforestation of regions along the upper reaches of the Yangtze River and the upper and middle reaches of the Yellow River; reconversion of farmland into forest or pasture in the eastern Tibetan Plateau, northern Xinjiang and eastern Inner Mongolia; and management of windy sand sources in Beijing and Tianjin. Figure 19a shows changes in vegetation cover from 2005 to 2008, demonstrating that these policies greatly increased vegetation cover in northeastern Inner Mongolia, Tibet and northern parts of central China from 2005 to 2008, and significantly improved the environment in these areas. Although vegetation cover is not obviously increased in Yunnan, Guizhou, Guangdong, Guangxi and Hainan, and even shows a decline in some regions, the significantly increased water resources make up for the shortage of vegetation cover. Figure 20a shows the change in rainfall during 2005–2008. It is one of the important influence factors leading to the water resources increased, the rainfall of these five provinces in 2008 were much higher than that in 2005. EQI declined in some regions of northwestern Urumqi, Xinjiang during 2005–2008. According to the BAI, the main vegetation of these regions was grass, and vegetation cover declined from 60% in 2005 to only 10% in 2008. This desertification caused the sharp deterioration of environment quality in these areas.

Figure 18 shows the change in EQI from 2005 to 2008. The significantly improved areas are mainly located in northwestern Xinjiang, eastern Qinghai, Fujian, Zhejiang and Jiangsu, suggesting that the Eleventh Five-Year Plan has been effective in achieving environmental protection. The change of vegetation cover during 2008–2010 is presented in Figure 19b. The rich water resources improved the environment qualities of Fujian, Zhejiang and Jiangsu. According to the China Statistical Yearbook for the years 2009 and 2011, the total water resources increased between 2008 and 2010 in Fujian Province (103.69–165.27 billion cubic meters), Zhejiang Province (85.52–139.86 billion cubic meters) and Jiangxi Province (135.62–227.55 billion cubic meters). Compared with 2008, the regions with slightly reduced water resources in 2010 were mainly in Yunnan, Guangxi, Tibet and northeastern Inner Mongolia. Meteorological data show that severe droughts occurred in the five provinces of southwestern China (including Yunnan, Guizhou, Guangxi, Sichuan and Chongqing) in 2010, with considerable impact on vegetation; in 2010, Inner Mongolia recorded the lowest rainfall and highest temperatures since 1961. The abnormal weather exacerbated the drought in Inner Mongolia and reduced vegetation cover. The five provinces of southwestern China received significantly lower rainfall in 2010 than 2005 (Figure 20c), while these five provinces and northeastern Inner Mongolia recorded markedly increased sunshine duration (Figure 20d).

## 5. Conclusions

This study examined eco-environmental status via national land use remote sensing data, MODIS NDVI data, and non-remote sensing information and statistical data. It modeled the Eco-environmental Quality Index (EQI) by extracting the Biological Abundance Index, Vegetation Index, Water Network Density Index, Land Degradation Index and Pollution Index, and evaluated and compared the environment status of China in 2005, 2008 and 2010. From the findings, the following points can be summarized: (1) In general, ecological environmental status is mediocre. Eco-environmental status in northwestern China is generally lower than in the southeast, with a ladder-like distribution. (2) Meteorological conditions, such as rainfall and sunshine show obvious effects on the distribution of eco-environment. There is a positive correlation between EQI and rainfall. Ecological quality of environment is higher in areas with more rainfall. The relationship between EQI and sunshine follows a quadratic curve. Ecological quality of environment is lower in areas with longer sunshine duration. (3) Across the eight economic regions, EQI is higher in those with higher population density and GDP density. However, when population density is saturated, the continuing increase of population and GDP density will lead to environmental deterioration. (4) The increase of EQI in Inner Mongolia and Tibet during 2005–2008 should be credited to the success of the policies outlined in the Eleventh Five-Year Plan, while the increase in southern provinces is due to higher rainfall. Desertification caused the sharp deterioration of ecological environment quality in some regions of northwestern Xinjiang. During 2008–2010, severe droughts occurred in the five provinces of southwestern China and Inner Mongolia, caused by environmental deterioration in these regions. 

In this study, land-use data were derived from visual interpretation of TM images, while the soil erosion data were collected in 1995. It is evident that updates to these data sets are very infrequent, and that they are not well suited to annual evaluation of EQI for China. Future work will therefore involve identifying suitable surrogate data sources. Secondly, we discussed four factors affecting environmental quality in China, but in practice EQI is influenced by many factors and the analysis in this article is therefore incomplete. Future studies will discuss additional factors such as energy consumption, and will also examine these factors in combination.
